# Inner speech and the body error theory

**DOI:** 10.3389/fpsyg.2024.1360699

**Published:** 2024-03-21

**Authors:** Ronald P. Endicott

**Affiliations:** Department of Philosophy and Religious Studies, Cognitive Science, North Carolina State University, Raleigh, NC, United States

**Keywords:** inner speech, cross-modal perception, illusionism, speech monitoring, consciousness

## Abstract

Inner speech is commonly understood as the conscious experience of a voice within the mind. One recurrent theme in the scientific literature is that the phenomenon involves a representation of overt speech, for example, a representation of phonetic properties that result from a copy of speech instructions that were ultimately suppressed. I propose a larger picture that involves some embodied objects and their misperception. I call it “the Body Error Theory,” or BET for short. BET is a form of illusionism, but the particular version I favor is a cross-modal illusion. Newly described here, my hypothesis is that the experience of inner speech arises from a mix of interoception and audition. Specifically, there is the detection of slight but well-confirmed activities in the speech musculature that occur during inner speech, which helps to transform representations of normal but quiet nonverbal sounds that inevitably occur during inner speech, from breathing to background noise, into a mistaken perception of inner speech. Simply put, *activities in the speech musculature* mix with *sounds* to create the appearance of *speech sounds*, which thus explains the “voice within the mind.” I also show how BET’s cross-modal system fits with standard information processing accounts for speech monitoring and how it accommodates the central insights of leading theories of inner speech. In addition, I show how BET is supported by data from experience-sampling surveys and how it can be empirically tested against its rivals.

## 1 Introduction

Inner speech is a fascinating phenomenon that has been commonly understood, from Plato until the present day, as a special kind of conscious experience, specifically, a voice within the mind (Plato, *Theatetus* 189e-190a; for a review of the scientific literature, Alderson-Day and Fernyhough, 2015; for an interdisciplinary collection, Langland-Hassan and Vicente, 2018).

Although there are many different views on inner speech, one recurrent theme is that the phenomenon involves a representation of overt speech, for example, a representation of its phonetic properties (Jackendoff, 2007; Langland-Hassan, 2018) that results from a copy of speech instructions that were ultimately suppressed (Carruthers, 2018; Loevensbruck et al., 2018; for the role of an efference copy in cognition more generally, see Miall and Wolpert, 1996; Shadmehr and Krakauer, 2008).

Since no overt speech occurs, the representation is false. I propose a larger picture of how that false representation arises within a cognitive system. I call it “the Body Error Theory,” or BET for short. BET is a form of illusionism (Dennett, 1991; Frankish, 2016). The particular version I favor is a cross-modal illusion. Newly described here, my hypothesis is that the experience of inner speech arises from a mix of interoception and audition. Specifically, there is the detection of slight but well-confirmed muscle activities that occur in the speech machinery during the inner speech, which helps to transform representations of normal but quiet nonverbal sounds that inevitably occur during inner speech, from breathing to background noise, into a mistaken perception of inner speech (for muscle activity detected by electromyography, see Livesay et al., 1996; Nalborczyk et al., 2017; for cross-modal perception, see Bertelson and De Gelder, 2004). Simply put, *activities in the speech musculature* mix with *sounds* to create the appearance of *speech sounds*, which thus explains the “voice within the mind.”

I begin by presenting some key aspects of the inner speech experience that are described in the scientific literature. I then develop BET and show how it fits with standard models of processing for speech monitoring, illustrating with the model developed by Ardi Roelofs (2020). I then turn to the evidential support, showing how BET explains the central data gathered by experience-sampling surveys (Hurlburt, 1990; Hurlburt and Heavey, 2006; Hurlburt et al., 2013). I also show how BET can be empirically tested, since it makes predictions that differ from currently popular theories that treat the experience of inner speech as a purely internal process, once triggered, without the external perception of speech activities and nonverbal sounds involved in BET’s cross-modal illusion. I then answer a potential objection, and I close with some general philosophical points about BET and how it might extend to other psychological phenomena.

## 2 The experience of inner speech

I begin by clarifying the phenomenon that BET aims to explain. Inner speech has been described in different ways, for example, as subvocal speech, verbal thought, or an internal monolog or dialogue. Inner speech also divides into a variety of sub-kinds, such as condensed versus expanded, voluntary versus spontaneous, normal versus hallucinatory, and varied versus ruminative in its content. However described and whatever kind, the phenomenon contrasts with other types of inner experience, such as inner seeing or visual imagery, and unsymbolized thinking or pure thought (for a larger list of inner experience, see Heavey and Hurlburt, 2008; Heavey et al., 2019). But, roughly speaking, unlike other forms of inner experience, inner speech involves *language-like experiences*.

As leading researchers Russell Hurlburt, Christopher Heavey, and Jason Kelsey state: “Most commonly it [inner speech] is experienced by the person as speaking in his or her own naturally inflected voice but with no sound being produced” (Hurlburt et al., 2013, p. 1477; see also Filik and Barber, 2011 for the point that the experience even includes one’s regional dialect).^1^ Also, based upon numerous interviews, Hurlburt, Heavey, and Kelsey find that subjects have an “unshakeable recognition that the speaking is inner rather than external” (Hurlburt et al., 2013, p. 1482). In short, subjects typically experience inner speech as *speaking their own public language in their own voice*, but in a *physically silent way* that is *inside the mind*. I will have something to say about the features of the typical inner-speech experience later. However, the present BET hypothesis aims to explain this central cluster of features associated with inner speech.

Note also that I refer to the *experience* of inner speech, which by itself does not imply that any relevant mental processing or mental representations themselves have the form of a public language. The processing might be in a symbolic brain code or mentalese, not in a public language. Or the processing might involve imagistic representations rather than language-like representations. In other words, the *content or meaning* of the relevant representations is one thing, whereas their *format or structure* within the brain is something else. The representations in the brain that underlie the experience of inner speech have the content of a public language—they are about aspects of one’s public language—even if their own format or structure is quite different (think of pixels representing a continuous line). For the purposes of BET, I leave the format question open.

To further clarify BET’s intended scope, some researchers focus on the experience of inner speech in terms of its production, an inner “speaking” (see Hurlburt et al., 2013), whereas other researchers include the experience of its reception wherein the mental utterances are “heard” in the mind (see Langland-Hassan, 2018). I intend BET to encompasses both inner speech and inner hearing.^2^ As should become clear later, BET’s advertised cross-modal illusion covers both matters of speech production (the incipient movements in the speech musculature) and matters of reception (those movements plus nonverbal sounds are perceived and then mixed to create cross-modal percepts).

Accordingly, inner speech is a complex cognitive mechanism with both speech production and speech reception systems that work together for various purposes, such as to strengthen memory (Baddeley and Hitch, 1974), bring first-order thoughts to consciousness by more palpable verbal clothing (Clark, 1998), aid in the imagination (Fossa, 2022), and other functions (for a wide range of functions, see Carruthers, 2011; Alderson-Day and Fernyhough, 2015; Barros et al., 2020).

Finally, not everyone experiences inner speech. Although the exact percentage is subject to debate, estimates vary considerably. For example, some estimates based upon interviews indicate that inner speech occurs in about 25% of the people interviewed (Heavey and Hurlburt, 2008). However, other studies based upon reading tasks indicate that the experience of inner speech occurs anywhere from 59 to 18 to 3% of those tested (Hurlburt, 2018; Moore and Schwitzgebel, 2018a,b).^3^ The discrepancies can be explained, in part, by different triggering conditions, the use of different methods, and individual differences among participants. But, based upon this kind of data, it is safe to say that a significant number of people do not experience inner speech. This means that they experience other forms of conscious thought without public words or sentences, say, as pure thought with no associated sensory qualities. In any case, BET applies only to those who experience inner speech. I will address later the possible forms of inner speech that BET leaves unaddressed.

## 3 BET

I propose to explain the experience of inner speech by the body error theory, or BET. Put in a general way, and the idea is this: *physical objects and qualities that exist in and around the body are both represented in the brain during inner speech and ultimately misinterpreted to be subjective objects and qualities of a voice within the mind because of their covert nature, close proximity to the brain, and the operation of specific cognitive illusions.* Let me begin by addressing the specific kind of illusion at issue.

### 3.1 Cross-modal illusions

I propose that inner speech involves a *cross-modal illusion*. To illustrate, consider the famous McGurk effect. Psychologists Harry McGurk and John MacDonald had subjects watch a video of someone speaking the *ba* sound in English, which they unsurprisingly heard as {ba}. But when the video with the same *ba* sound was mixed with a visual presentation of the same face now articulating the *ga* sound, this caused subjects to hear something different, a new percept {da} instead, or sometimes a combined percept such as {bagba} (McGurk and Macdonald, 1976). This is a visual-changing-auditory case. But there is also the reverse. For example, Ladan Shams, Yukiyasu Kamitani, Shinsuke Shimojo found that a single visual flash, when accompanied by multiple auditory beeps, is then visually perceived as multiple flashes (Shams et al., 2000; for a previous auditory-changing-visual case, see Radeau and Bertelson, 1974).

There are also auditory-changing-tactile cases, such as the parchment-skin illusion whereby a high-pitched and rough sound presented when subjects rub their hands together then modifies the resulting tactile sensations and makes the hands feel drier or paper-like (Jousmäki and Hari, 1998). The moral is that the brain contains mechanisms for multisensory integration, systems for a kind of “sensory mash-up” that results in fused or altered percepts. Accordingly, I propose that inner speech involves a cross-modal illusion akin to a tactile-changing-auditory case. Specifically, BET is an interoceptive-changing-auditory type of cross-modal illusion, mixing sound and bodily activity.

### 3.2 Sensory inputs for BET’s cross-modal illusion

I start with the auditory inputs. There are normal physical sounds that occur in and around the body during inner speech, which is to say, pressure waves caused by various things from objects and movements in the local environment to the soft sounds that emanate from one’s breathing. Although one may not be consciously aware of them via auditory processing, at least not without some focus of attention, I assume that these physical sounds are nevertheless detected and represented in the brain. For example, although public speech measures around 60 decibels, an exhale from breathing measures around 10 decibels, which is well within the range of detection by a normal human system for hearing.

The second kind of input involves slight but measurable activity in the speech musculature that occurs during inner speech (for data from electromyography, or EMGs, the pioneering work is Jacobson, 1931; see also Sokolov, 1972; Livesay et al., 1996; Nalborczyk et al., 2017; and a summary in Geva, 2018). The most oft-cited data involves electrical activity in the orofacial muscles, including muscles for articulation, for example, in the lips during a mental recitation of the Pledge of Allegiance (Livesay et al., 1996) and during the experience of verbal rumination or repetitive inner speech (Nalborczyk et al., 2017). However, the relevant muscle activity is not confined to mere electrical activity. Rather, it also involves changes in the tension of the muscles and their actual movements, although they are quite small in comparison with overt speech, and they produce no sounds.

Consider what I call *motion-detection* experiments, in contrast to the standard *electrical-detection* experiments provided by EMGs. These involve various technologies, for example, a mechanism that detects vibrations on the tongue during inner speech (Sokolov, 1972), a pneumograph on the chest to measure changes in the circumference of the thorax during respirations for both outer and inner speech (Conrad and Schönle, 1979), and ultrasounds for the vocal tract combined with a video camera for the lips to detect position and movement during both overt and inner speech (Florescu et al., 2010).

Thus, psychologist Alexander Sokolov (1972) placed a pickup on the anterior of the tongue, responding to vibrations that occurred both lengthwise and across the tongue. The device then transformed the vibrations into electrical pulses, creating a kymogram that displayed the pulses in a graph. Sokolov then tested college students under three conditions that evoked the experience of inner speech: while they solved a simple math problem, read a text silently, and listened to a text, all of which he compared to a baseline control when these cognitive tasks were not performed. The results were quite positive, indicating that, to quote Sokolov: “the execution of mental operations … is accompanied by well-defined tongue movements,” although they are small “micromovements” when compared to public speech (Sokolov, 1972, pp. 161–2).

Also, these micromovements are specific to the muscles that correspond to the kind of experienced utterances that occur during inner speech. Utilizing EMGs, psychophysicists Frank McGuigan and Andrew Dollins found that the lips are active in a way that is appropriate for a bilabial pronunciation when processing the letter “P” but not “T” during a visual reading task, whereas the tongue is active in a way that is appropriate for an alveolar pronunciation when processing the letter “T” but not “P” (McGuigan and Dollins, 1989). They concluded that “the speech musculature covertly responds systematically as a function of the class of phoneme being processed” (*ibid.*, p. 19). Given this systematic correspondence, I think the small activities and movements are accurately described as *incipient speech activities*.

Now, there are different views about exactly how one should understand these incipient speech activities. For example, some view them as covert but cognitively driven linguistic behavior, whereas others view them as a mere residual effect of commands from the brain to override linguistic behavior. Or, again, some believe that there is a strong correlation between the stated incipient speech activities and the phenomenon of inner speech, whereas others believe that the two are only weakly correlated. I will address these issues in the sections that follow. But with the two factors in hand, I am now in a position to sketch a specific hypothesis.

### 3.3 The BET illusion and its information processing

The specific hypothesis is this:

[BET] *incipient speech activities* are represented via interoceptive channels at a sensory stage of perception during inner speech, and *sounds* are represented via auditory channels also at a sensory stage of perception during inner speech. Both of these representations then concurrently feed into downstream mechanisms for multisensory integration, creating fused percepts of *speech sounds*, pace an interoceptive-changing-auditory case of a cross-modal illusion, which thus explains the experience of inner speech.

To put the idea like the McGurk effect, start with a linguistically unstructured percept {ahh} that was caused by an exhale *ahh* during inner speech. But the brain simultaneously knows that there is a pattern being tapped out in the speech machinery that has long served public speech, say, the tensing of muscles that would serve the phoneme *da*. This activates cross-modal mechanisms that create the fused and linguistically structured percept {da}. Or, put more simply, *incipient speech activities* and *sounds* become mixed into *speech sounds*, which thus explains the “voice within the mind.”

Let me add some initial points of clarification. First, the error need not make people actually *believe* what is false, only that it *seems* or *appears* in that false way (think of the Müller-Lyer illusion wherein lines of equal length *appear* otherwise even when one knows they are equal). Second, BET only requires the *detection* of the stated stimuli rather than their full *conscious perception* (for detection versus conscious perception with respect to interoception, see Köteles, 2021, chap. 4). Indeed, until there has been a focus of attention, subjects are largely unaware of the incipient speech activities and sounds as such, although they lead to the advertised illusion in consciousness. Third, I speak mainly of “interoception” for the activity in the speech musculature, but one may also speak of “proprioception” and “kinesthesia,” although the terms are often used with a different emphasis (a sense of the body versus a sense of something inside the body).

Fourth, although the proffered cross-modal illusion might seem unusual, mistakes involving sound are quite common. Some involve a simple *mislocation*, for example, someone hearing the voice of a ventriloquist as if it belonged to a dummy perched close by. Other mistakes involve a *change* in how the sound is perceived, such as when I mistook my dog’s snoring at the foot of my bed for my child faintly talking in the next room. The postulated interoceptive-changing-auditory case for BET involves both kinds of mistakes. One mislocates sound that is in and around the body as something inside the mind, and there is a change in how the sound is perceived, from mere sound to one structured by the activity in the speech machinery.

Let me now sketch an account of BET’s information processing. I favor a conservative extension of a family of views about speech monitoring. To begin, BET is compatible with several current models of speech monitoring that involve *two groups of systems*: one with an internal loop of processes and another with an external loop of processes that involve the production of speech and its perception for the purposes of monitoring that speech.^4^

The internal systems are commonly associated with inner speech, meaning the purely internal loop of systems that construct a linguistic representation that is then copied and sent back into the system before any motor activity (a “feedforward” rather than “feedback” system). The system then makes a prediction based upon that efference copy that can be compared to the actual results that are perceived through the external channels, thus allowing the system to make adjustments in real time. As psychologists Ben Alderson-Day and Charles Fernyhough describe the process: “inner speech largely serves to support error monitoring in speech production, whereby utterances can be inspected and corrected via an ‘internal loop’” (Alderson-Day and Fernyhough, 2015, p. 941; for a classic statement that inner speech plays this role in speech monitoring, see Levelt, 1983; and for some currently popular models that involve the stated external and internal systems just described, see Pickering and Garrod, 2013; and Roelofs, 2020).^5^

Now, there is typically no overt speech during the conscious experience of inner speech. As Hurlburt, Heavey, and Kelsey note, based upon the data they collected: “most external speaking is not accompanied or preceded by inner speaking” (Hurlburt et al., 2013, p. 1484). Hence, the external channels that would normally serve overt speech are available to produce and then detect the silent but incipient speech activities that occur during episodes of inner speech. Accordingly, BET describes the same internal and external loop of systems described by familiar models of speech monitoring, only with the external system turned down but still active to produce the more covert product of incipient speech behavior. In other words, the external but slight activity in the speech muscular still occurs, even though the overt speaking does not.

**Figure 1:**
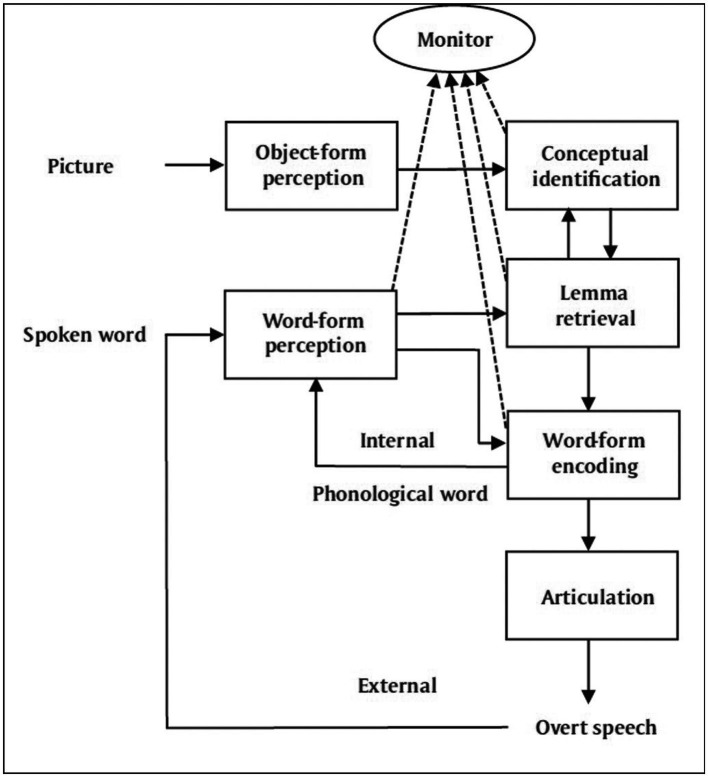
Internal + External Loop model of speech monitoring (Roelofs, 2020, p. 3).

**Figure 2:**
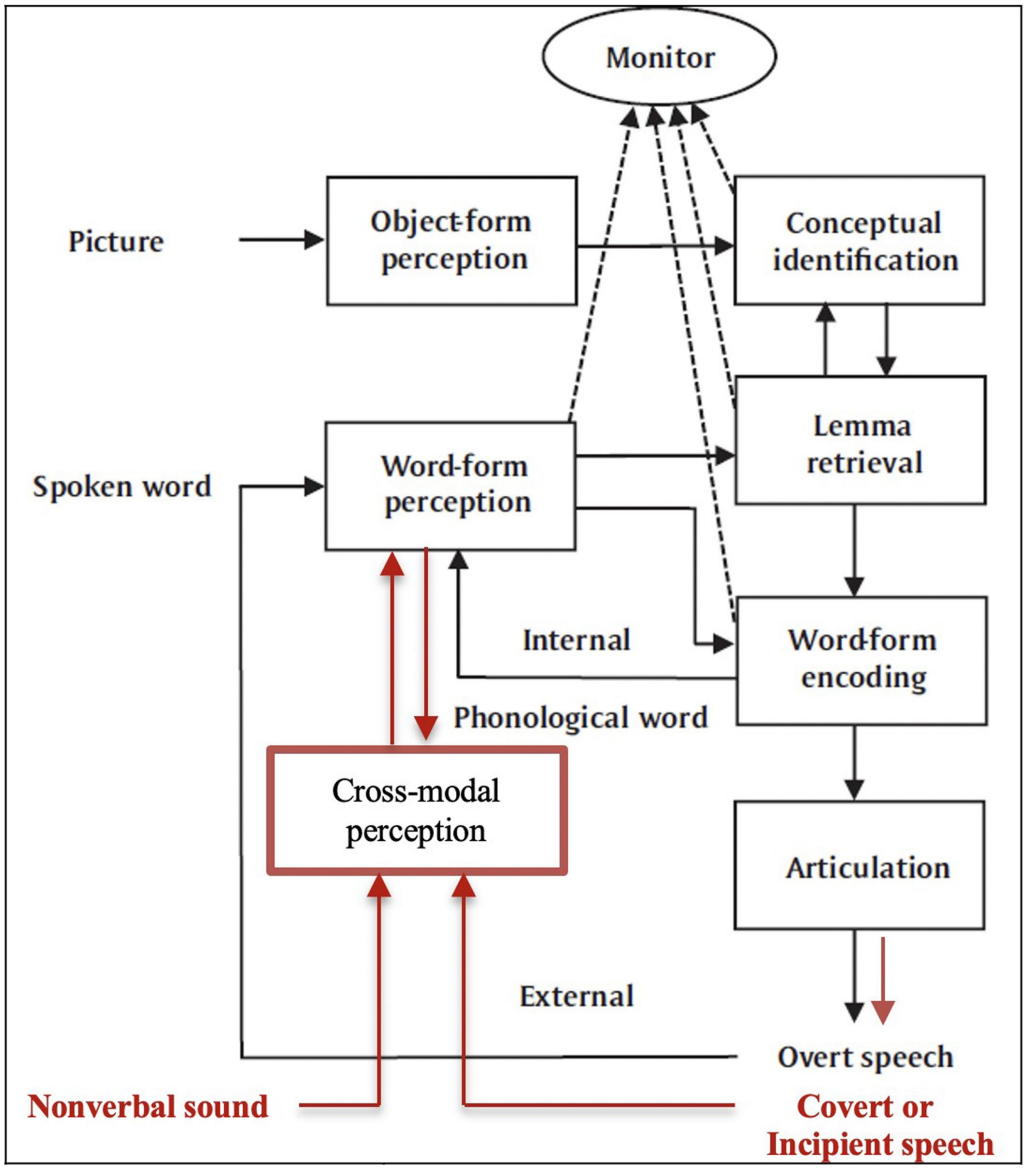
The BET modification.

To illustrate, I present Ardi Roelofs (2020) schematic diagram of his account of speech monitoring below, in Figure 1, along with my suggested expansion for BET, in Figure 2, with additions highlighted in red.

Regarding the model depicted in Figure 1, Roelofs says that the subject sees a picture and says “cat,” which he describes in this way: “In naming a picture, the phonological word representation of the picture name is fed into the speech comprehension system (the internal loop), which also processes the overtly articulated picture name (the external loop)” (Roelofs, 2020, p. 3).^6^ Regarding the expansion in Figure 2, suppose that the subject sees a picture of a cat, say, during a silent reading task. By BET, even though an overt utterance of “cat” is not initiated, the appropriate internal processing produces the covert speech activities in their stead, which are then detected as part of the usual perceptual feedback loop and then mixed with incoming sound to create the cross-modal percept {speech sound “cat”}.

Thus, the experience of saying “cat” within inner speech is a consequence of the stated processes. So (i) the motor control for overt speech can be dampened in order to produce slight activities in the speech musculature. As well, (ii) a system for cross-modal perception is connected to the system for word-form perception, which enables the cross-modal perception system to draw upon information about the formation of words that had been sent through the internal loop, thus helping with the task of interpretation for the incoming speech activities and reflecting the effects of expectation created by the instructions. The cross-modal system then mixes the felt speech, so interpreted, with the incoming sounds to produce the fused representations of speech sounds.

Finally, I assume that, as a form of muted speech behavior, the incipient speech activities described in Figure 2 play a role analogous to the function of overt speech in the process of speech monitoring in Figure 1. They are physical inputs for the relevant perceptual processing. Also, the entire mechanism contributes to the function of still larger systems associated with inner speech, such as rehearsal for memory, planning for action, bringing first-order thoughts to consciousness, and others mentioned earlier. Indeed, one can now see why BET includes functions for both inner speech and inner hearing since it includes systems for both speech production and speech reception through perception.

Yet the proposed model for BET’s information processing says nothing about consciousness, at least not directly, and there is no consensus about the best way to understand consciousness itself. Nevertheless, I think one may add the kind of processing required by leading theories of consciousness, say, stipulating that the monitor in Figure 2 sends information out in a globally broadcast way, to a workspace for consciousness, or to a system that contains higher-order thoughts about that information (for surveys, see Seth and Bayne, 2022; Van Gulick, 2022, sec. 9).

## 4 Supporting data and how BET can be empirically tested

### 4.1 Scientific data explained

I think BET explains a large range of data for the experience of inner speech. This includes numerous cases collected by the descriptive experience-sampling method, or the DES (Hurlburt, 1990, 2011; Hurlburt and Heavey, 2006; Hurlburt et al., 2013).^7^ Hurlburt, Heavey, and Kelsey state that the following features are contained in “the most frequent and ‘center of the target’ examples of inner speaking” (Hurlburt et al., 2013, p. 1482). Consider their first three features:

[1] The person apprehends him or herself to be speaking meaningfully without producing any accompanying sound or appreciable bodily (throat, diaphragm, etc.) movement.

[2] The speakings are generally apprehended to be in the person’s own naturally inflected voice, in the same rhythm, pacing, expressivity, tone, hesitations, and style as external speaking (sometimes with a greater range of expression than external speaking).

[3] The experience is typically apprehended to be just like speaking aloud in the sense that people who report innerly speaking are typically at a loss to identify any aspect in which the experience differs from externally speaking other than their immediate and unshakeable recognition that the speaking is inner rather than external (confusion about whether something is innerly spoken or spoken aloud is very rare) (Hurlburt et al., 2013, p. 1482).

I mentioned these three features at the outset, although in a slightly different order. By the present list, inner speech is typically experienced by subjects as [1] *speaking* in a *physically silent way*, [2] *with their own public language in their own voice*, and [3] *inside their own minds*. Now, BET explains these features in a straightforward way. Subjects experience the phenomenon as *silent* because the inner activities plus the incipient speech activities *are* silent (they produce no sound) and because the subjects do not consciously make the connection with normal nonverbal sounds that occur, which their brains then mix with the perceived speech movements, pace the cross-modal illusion. That accounts for the datum [1].

Parenthetically, in order to guard against a possible misunderstanding, notice that Hurlburt, Heavey, and Kelsey say that subjects apprehend themselves to be speaking “without producing any accompanying sound *or appreciable bodily (throat, diaphragm*, etc.*) movement*” (Hurlburt et al., 2013, p. 1482, my emphasis). One might worry that the latter point conflicts with BET’s incipient speech activities. But, to the contrary, BET only claims that the slight and incipient speech activities are typically sensed but not consciously acknowledged, befitting the status of inner speech as an illusion that arises from those activities. As such, the relevant covert activities are not “appreciable” from the subject’s perspective, even though they contribute to the overall illusion.^8^ That is, not being appreciable is perfectly consistent with their covert status.

Thus, to continue with the list of features, subjects also experience themselves *speaking with their own public language in their own voice* because they are really producing incipient speech activities from their own public speech-and-monitoring system, although its outputs are modulated downward for a covert product. That explains the datum [2]. Furthermore, subjects think that the phenomenon is *inside their minds* because they know that there is no corresponding overt speech that they are producing at the time in question and because they are typically unaware of the true sources that are outside their minds, namely, their incipient speech activities plus soft nonverbal sounds that their brains mix together to complete the illusion of their own “speech sounds.” That explains the datum [3].

However, let me add that subjects *typically* identify the experience as something inside their minds, but not always. As Hurlburt, Heavey, and Kelsey also note, some people report that inner speech occurs “primarily in their chest or midsection” (Hurlburt et al., 2013, p. 1483). The researchers do not comment on this any further. But the reports make perfect sense, given the BET hypothesis, since the activities in the speech machinery are not *confined* to the musculature in the mouth and throat. The human brain can also detect the activities in the diaphragm and lungs, including the irregular breathing pattern that occurs during both outer *and* inner speech, namely, shorter and harder breaths during inhalation, then longer and softer breaths for exhalation (as confirmed in Conrad and Schönle, 1979).^9^ BET’s embodied activities thus explain both the common and uncommon experiences of inner speech.

I now turn to another group of features that Hurlburt, Heavey, and Kelsey describe, which I combine together as items relevant to the *linguistic content* of inner speech experiences:

[4] Inner speakings are generally but by no means always in complete sentences. Five of our six original examples are in sentences (e.g., “I do not want to go”). Sometimes the speakings are just one or a few words (e.g., “Thai food!”). The significance of the words, whether in complete sentences or condensed, is generally understood … Inner speaking is generally in the same kinds of words that the person would use in external speech, including the same kinds of quasi-worded expressions (e.g., “Ugh!” or “Oof!”) as external speaking (Hurlburt et al., 2013, p. 1482).

This feature is relevant to the *language-like aspects* of the inner-speech experience referred to in [1] through [3], namely, that one experiences something like the complete and condensed sentences of one’s public language. Moreover, BET explains this feature because of the extra-abundance of resources displayed by its additions to speech monitoring during episodes of inner speech. For example, why does one experience the mental sentence “I do not want to go”? Because, by BET’s account (see Figure 2 in sec. 3.4), an internal copy of instructions for that sentence, replete with syntactic, semantic, and phonological information, is processed within the internal loop of systems. This copy is then compared to the inputs from one’s external loop of perceptual systems that are busy tapping out a schematic pattern of that sentence in response to the real but dampened activities and micro movements in the body that correspond to the beginnings of the phonetic parts of the very sentence in question. That explains the datum [4]. Indeed, according to a longer story I will tell later on, there is a division of labor in the explanation such that the external perceptual processes account for the phenomenal qualities of the language-like experience, whereas the internal processes account for its more abstract information.

In any case, Hurlburt, Heavey, and Kelsey discuss more than points [1] through [4]. For example, they make the distinction between reports for inner speech versus inner hearing, which I have already addressed (BET covers both since it includes systems for both speech production and speech reception through perception). I grant that there might be some phenomenon that falls under the category of inner speech which BET does not address, for example, a kind of unworded and non-phenomenal kind of thinking that I will return to in my concluding section. But I think I have said enough to indicate how BET explains the central data gathered from the experience-sampling surveys for inner speech.

### 4.2 How BET may be tested against competing theories

BET is also a substantial empirical hypothesis that makes predictions which can be tested against rival theories. To illustrate, consider the currently popular *mental simulation view* of inner speech, or SIM for short. On this kind of view, inner speech is a mental simulation of the motor activity for overt speech. In light of my earlier discussion of speech monitoring, simulations are tied to the processes whereby instructions for speech production are sent through the inner loop of systems (see Figure 1 in sec. 3.4). A simulation also provides a paradigm for what some call the “activity” view of inner speech (Martinez-Manrique and Vicente, 2015, p. 1), otherwise described as “the actual speech view” (Gregory and Langland-Hassan, 2023, sec. 1). But the kind of activity so described is an internal mental activity, and the actual speech in question is a supposed act of mental speech.

Now BET is compatible with mental simulations, for they can be part of how a BET-style system operates, running a simulation based upon an efference copy in the inner loop that is then compared to the percept constructed through the outer loop of perception, only now what is perceived is a modulated covert behavior (see Figure 2 in sec. 3.4). However, advocates of SIM often express additional assumptions that run contrary to BET. I call one the “blocking assumption,” meaning that the motor commands for speech are blocked, and the motor-control systems are taken offline.

Thus, neuroscientists Ladislas Nalborczyk and colleagues describe their motor simulation view according to which “the motor execution is blocked” (Nalborczyk et al., 2017, p. 53). Similarly, when describing his simulation view, the philosopher Peter Carruthers says that inner speech involves “auditory images that result from offline activation of instructions for producing speech” (Carruthers, 2014, p. 149). Or again, describing the simulation in terms of a “sensory forward model” that transforms a copy of the motor command into a prediction of an outcome for overt speech that does not occur, Carruthers says that inner speech “is just a sensory forward model in auditory code processed by activated (but not executed) speech actions” (Carruthers, 2018, p. 35, my emphasis).

BET presents a different picture whereby the process for inner speech is *online* and active, resulting in a covert form of incipient speech. Indeed, the well-confirmed activities in the speech musculature present a problem for the contrary blocking assumption. As Hélène Loevensbruck and colleagues observe when developing their own simulation view: “If inhibition prevents motor acts from actually being executed, then the neurophysiological activity in the muscles must be explained” (Loevensbruck et al., 2018, p. 142). Loevensbruck and colleagues go on to offer such an explanation, extending some previous remarks by neuroscientists Marc Jeannerod and Jean Docety regarding motor activity and imagined actions:

We suggest that motor commands might be emitted, together with inhibitory signals blocking articulatory movement. This speculation is in line with Jennerod and Decety’s (1995) description of action imagery. According to them, during mental simulation of action, “it is likely that the excitatory motor output generated for exciting the motor action is counterbalanced by another parallel inhibitory output. The competition between the two opposite outputs would account for the partial block of the motor neurons, as shown by residual EMG recordings and increased reflex excitability (p. 728)” (Loevensbruck et al., 2018, p. 142).

Call this “the deflationary explanation” for incipient speech behavior, which one may add as a charitable interpretation of the blocking assumption—*the covert activities in the speech musculature are not entirely blocked, but what remains is a mere residual effect*.

Now, I think some remarks in the above explanation are perfectly acceptable. For example, the suggestion that motor commands are emitted together with inhibitory signals is an insightful way to account for the activity at issue, even a whole range of modulated speech activity (more on that later). Even so, the remark quoted from Jennerod and Decety that the excitatory motor output is “counterbalanced” by a parallel inhibitory output is an overstatement since the motor commands have not been entirely negated or neutralized, and the inhibitory output is not an equal but opposite effect. Also, although one may call the activity a “residual” effect from two opposing streams of signals, one may say with equal justice to the facts that the excitatory motor signals are simply *stronger* than the signals to suppress them. The motor commands win slightly.

Certainly, the mere fact that there is a mechanism for excitatory and inhibitory signals does not address BET’s perceptual claim that the slight activities in the speech musculature which result from the operations of that mechanism are, in turn, perceived via interoception as part of the feedback for speech monitoring. The mechanism might explain why there is a small level of activity in the speech musculature (inhibitory signals dampened the effect of the motor commands). *It does not explain why the small level of activity that remains either is or is not detected by the brain’s interoceptive network*.^10^

So, the real issue is whether those slight activities play a continued functional role within the mind’s processing of inner speech beyond being an effect of excitatory versus inhibitory signals. Call BET’s particular sub-thesis about those further processes and functional roles “the external perceptual claim.” SIM, or the common package of SIM plus the blocking assumption/deflationary explanation, denies BET’s perceptual claim by maintaining that there is nothing functionally interesting that occurs during inner speech beyond the slight activities in the speech musculature—they are a “mere residual effect” that does not provide inputs for interoception as part of the feedback for speech monitoring.

In sum, there is a stark contrast between BET’s external perceptual claim for the incipient speech activities and SIM’s deflationary interpretation of the same activities, and this difference constitutes a fact that can be tested. Indeed, the same is true for BET versus any purely internalist theory, which, like SIM, does not share BET’s perceptual claim. For example, BET contrasts with standard developmental views whereby inner speech is the end result of a transition from overt speech to the whispers of private speech to a purely internalized process (Vygotsky, 1934/1986; Vissers et al., 2020). The revisionary BET maintains, instead, that inner speech is not *completely* internalized, given the role of perception for things in and around the body. In any case, I present the different predictions below, adding INT for other purely internalist theories quite generally (Table 1).

**Table 1:** With different predictions from BET, SIM, and INT regarding the external perception of the slight activity in the speech musculature during inner speech.

	External perception	Internal processes
BET	Yes	Yes
SIM	No	Yes
INT	No	Yes

I would be surprised if the pertinent activities in the speech musculature fell below the threshold of detection by human interoception. The tongue and lips are densely packed with assorted kinds of receptors to detect such things as taste, temperature, pressure, movement, and stretching (for the lips, see Martín-Cruces et al., 2023). They are even more sensitive than the fingertips. For example, in a spatial gradient experiment, the lips were able to detect groove widths of 0.51 mm, the tongue at 0.58 mm, and the fingers at 0.94 mm (Van Boven and Johnson, 1994).

Furthermore, there are other reasons to expect a positive result for BET’s perceptual claim. For example, there is already a successful human–computer interface that illustrates how the slight activities in the speech musculature can be perceived via a simulated interoception. Specifically, computer scientist Arnav Kapur has developed an AI system interface, *AlterEgo*, that simulates the human inner speech system by reading the electrical signals in the orofacial muscles during a subject’s inner speech by means of noninvasive surface EMGs in a wearable mask, drawing upon data of previously collected vocabulary from test subjects who engaged in inner speech. *AlterEgo* then provides a translation of the intended words that is 92% accurate and sends the result via an audio output to the brain by means of a small microphone whose slight sounds are conducted through the bones in the skull and ear, closing the feedback loop to the brain (Kapur et al., 2018).

The important point, for present purposes, is that *AlterEgo* proves that the slight activity in the speech musculature *can* function as inputs for perceptual feedback involving representations of those muscle movements. These inputs then feed into the relevant computational processing. Granted, a computer simulation is not proof that the human brain *does* operate in this fashion. But the success of *AlterEgo* is proof that the activity in the speech musculature is *readable* in the way BET requires, and contrary to any strong deflationary interpretation that would deny its potential informational value for a perceiving brain. Indeed, I assume that the unaided human brain is able to read its own covert muscle activity just as well, if not better, for the brain has an advantage—*AlterEgo* is limited to information gleaned from activity at the surface of the face and neck, whereas the human brain has a network of interoceptive channels that reach deep into the muscles and over a greater range of relevant areas in the body.

Finally, BET’s perceptual claim could be easily tested by running brain scans on the appropriate interoceptive channels during inner speech in order to determine if they are active or by simply duplicating the slight movements with an instrument placed on the tongue or lips in order to determine if subjects feel those movements.^11^ In fact, there are various experiments by which neuroscientists have stimulated the tongue and recorded their reception in the brain (see Sakamoto et al., 2010). I do not know of any experiments that match the small values exhibited during inner speech. So, until this kind of confirmation or disconfirmation is carried out, it is premature to draw any conclusions with confidence. But BET remains an interesting and testable hypothesis.

## 5 An anticipated objection and a defense of BET

I mentioned earlier that some believe there is a strong correlation between the incipient speech activities utilized by BET and the experience of inner speech, whereas others believe that the two items are only weakly correlated. Let me now address why some believe the latter.

### 5.1 The Smith experiment

Some researchers believe that any strong connection between inner speech and actual motor behavior has been refuted by an experiment wherein medical scientist Scott Smith had curare administered to himself but was conscious while on an artificial respirator (Smith et al., 1947). For example, Loevensbruck and colleagues say this: “The extreme view that inner speech requires actual movement has been refuted by Smith et al. (1947) who showed that temporary paralysis induced by curare did not prevent verbal thought” (Loevensbruck et al., 2018, p. 135). Similarly, although they have behaviorism specifically in mind, Gary Oppenheim and Gary Dell express the alleged refutation directly in terms of the slight movements in the speech musculature:

One of the earliest ideas about thinking was that it is nothing more than inner speech, a weakened form of overt speech in which movements of the articulators occur, but are too small to produce sound (Watson, 1913). A remarkable experiment by Smith et al. (1947) demonstrated that this idea was false. Abolishing any trace of articulation through curare-induced total paralysis (requiring a respirator!) did not impair the participant’s (Smith, himself) ability to think or understand his colleagues’ speech (Oppenheim and Dell, 2010, p. 1147).

Now, granted, behaviorism *is* false. Behaviorism posited a simple association relation between the appropriate stimuli for inner speech and the stated covert behavior that is inadequate, as the success of subsequent cognitive theories has abundantly shown. Accordingly, BET is a full cognitive theory that adds representations, speech monitoring, and cross-modal perception to covert behavior. Indeed, although previous cognitive theories of inner speech did not appeal to such things as speech monitoring and cross-modal perception, they did incorporate the covert activity—Sokolov’s neurocognitive approach to inner speech (Sokolov, 1972), McGuigan’s bio-cybernetic approach to inner speech (McGuigan, 1966, 1967, 1994), and assorted other motor theories of inner speech (for a review, see Galantucci et al., 2006).^12^ But the question remains: Is the correlation between BET’s incipient muscle activities and inner speech refuted by the Smith experiment? No.

### 5.2 The irrelevance of the experiment for inner speech

To begin, the Smith experiment only refutes a correlation between thought and *overt movements*. Thus, McGuigan observed that curare does not remove all the relevant bodily activities. After citing his earlier work, McGuigan says:

Hence, although there are no overt responses in the curarized state, important minute (covert) responses still occur. Some researchers did monitor EMG when they used curare during autonomic conditioning, but the sample electromyograms offered show covert behavior of perhaps as much as 20 microvolts in amplitude in presumably paralyzed animals. Such covert behavior could have important consequences because it and its consequent feedback may be sufficient to maintain cognition …” (McGuigan, 1994, p. 340).

Not only is the use of curare compatible with slight activity in the musculature, as shown by animal experiments, but the experiment on Smith did not utilize an EMG for Smith’s speech musculature or any other technology in order to show a lack of covert activity. There was only an “electro-encephalogram” for his brain that was not specific to inner-speech areas, plus an “electrocardiogram” for his heart (Smith et al., 1947, p. 9). So, the incipient speech activities might have occurred. The experiment was not designed to detect them or their absence.

Moreover, the original study by Smith and colleagues was only relevant to *conscious thought per se*, not inner speech or verbal thought (recall that other forms of conscious thought exist, such as wordless thought or pure thought). Certainly, the authors never mention inner speech in their published report, using terms for conscious thought and experience quite generally, such as whether there were “cerebral effects” under curare (Smith et al., 1947, p. 1) or whether curare has the property to “depress central mechanisms” (*ibid.* p. 2), or whether “pain” can still occur in the patient (*ibid.*, p. 3), all regarding their expressed concern as medical scientists about using curare “in anesthesia” (*ibid.* p. 1).^13^ Most relevant, the authors did not impose any method for testing the presence of inner speech as opposed to thought in general or more abstract thought in particular. For example, they did not provide explicit instructions for verbal rumination or ensure that Smith engaged in a problem-solving task with steps that would require working memory and its phonological system tied to inner speech. So, the experiment was relevant to “thinking” (Anderson, 2015, p. 364), but not inner speech.

Granted, Oppenheim and Dell are quite right to point out that Smith’s ability to understand his colleagues’ speech was unaffected. However, this is not relevant to the question of whether Smith experienced inner speech during the experiment. Understanding the overt speech of another individual is one thing; experiencing inner speech is quite another. To the point, Oppenheim and Dell seem to have assumed that Smith’s language comprehension implies the experience of inner speech. But that is false. For example, recall that a significant number of people do *not* experience inner speech. Yet, this significant number of people have not thereby lost their ability to interpret and produce speech.

Of course, there is *some* connection between language comprehension and the experience of inner speech. What is arguably true is that the experience of inner speech implies language comprehension. But that is the converse of the claim at issue. Oppenheim and Dell claimed that Smith’s language comprehension implies inner speech, which is the other way around. In any case, I have argued that, for all the experiment shows, Smith might not have engaged in inner speech while his (overt) behavior was suppressed. Indeed, Smith might have been one of the significant numbers of people who do not experience inner speech at all, even though his capacity to comprehend language remained intact and even though his overt behavior was suppressed. I think there are other potential problems for BET, which I lack the space to consider. But the famous Smith experiment is not a problem.

## 6 Concluding philosophical reflections, limits, and suggestions for future directions

### 6.1 Philosophical themes

I mentioned earlier that BET is a form of illusionism. Indeed, BET weaves together three current themes in cognitive science: illusionism for cases when there is a misleading conscious perception as opposed to purely cognitive errors or delusions; a representational theory of consciousness whereby conscious experience, illusory or not, is explained by the subject having representations with the content supplied by the objects so represented; and a modest embodiment thesis whereby some objects in and around the body are represented in conscious experience. Let me address these assumptions.

To begin, philosophers and scientists use “illusionism” in different ways regarding conscious experience, for example, to deny that consciousness is ineffable, intrinsic, or private (Dennett, 1991) or that there is a continuous stream of consciousness (Blackmore, 2002). Keith Frankish (2016) offered a helpful distinction between “strong” illusionism, which denies the existence of consciousness, versus “weak” illusionism, whereby consciousness exists but not with all the properties commonly attributed to it. I offer BET as a weak version. There is something fruitfully described as “inner speech,” but it lacks some of the properties commonly attributed to it. What is lacking?

According to BET, to speak of a pure “inner” speech is not quite accurate, since the phenomenon is not entirely inside the mind because of the involvement of external perception for the incipient speech activities and sound. Granted, BET’s covert speech activities are *directed* by the mind, but that does not make them *inside* the mind—no more than my walking is something inside my mind because it is directed by my mind. Indeed, the BET illusion is precisely that people tend to mistake real-world inputs for something inside the mind. Even so, one may continue to call the phenomenon “inner” speech, but with the qualification that it is not entirely so.

I also cast BET in terms of a representational theory of consciousness, meaning roughly that a conscious experience of something is explained by having a mental representation of that thing. For example, one has a conscious visual experience of a red rose because one has a representation developed through visual perception of the content of a red rose. By BET, there are representations of one’s public language, such as the cross-modally mixed {speech sounds}, as well as representations acquired through interoception {incipient speech activities} and audition {sound}. I favor a version of a representational theory of consciousness whereby the content of a representation is relativized to sensory modalities and allowed to reflect objects and qualities that exist beyond the brain and into the environment (for different versions, see Chalmers, 2010). But I allow that a representation may involve more than one sense modality, as in a cross-modal case.

As for the semantics, there is no consensus about the correct theory of meaning, reference, or the content of representations more generally. Moreover, there are assorted kinds of representations—perceptual representations, abstract concepts, and pure logical concepts—and some theories are better suited for one kind of representation rather than another. However, BET builds the stated illusion out of representations in sensory stages prior to a full conscious perception of the illusion, and for the sensory representations, I am inclined to accept a kind of teleological causal theory whereby a representation, say, {red} from the visual system, has the content *red is present* because, in part, the visual system has the function to causally produce that representation in response to the presence of red (see Neander, 2013).^14^ Thus, the representations in the interoceptive and auditory channels are functioning according to their biological design by being responsive to activities in the body and sound in the environment, respectively (e.g., the sensory representation{incipient speech activities} denotes *incipient speech activities* because the latter causes the former in accordance with the design of the speech monitoring system under the condition that its outputs are dampened—overt speech is absent under those conditions and thus not designated).

Of course, representations with no apparent referent raise special difficulties, like the illusory {speech sounds} when no speech sounds are uttered. Some posit an unactual object or an intentional inexistent as the referent in cases wherein represented objects do not seem available (see Lycan, 1996). Nevertheless, {speech sounds} are a complex construction, and by BET, the key representations which ultimately explain the experience of inner speech refer to actual felt and heard objects, respectively. Hence, BET is committed to the idea that any content of the false {speech sounds} that factors into the experience of inner speech is built from the true sensory percepts {incipient speech activities} and {sounds} with their contents that are grounded in the properties of real and available physical objects through perception, much like the content of the {gold mountain} is derived from the content of {gold} and {mountain}.^15^

Put differently, beyond the sensory materials given by the components that ground the experience, the additional false {speech sounds} involves, *ex hypothesi*, only non-phenomenal operations, like an inference in pure logic that has no phenomenal feel: “if ({incipient speech activities} and {sounds}) then {speech sounds}.”^16^ In this way, the sensory content explains the phenomenal content, with no remainder left over. There is thus a division of labor in the explanation, which I alluded to earlier, namely, that the representations generated by external perceptual processes account for the phenomenal qualities of the language-like experience for inner speech, whereas the representations generated by internal processes account for the additional abstract information. One can have pure thoughts about language. But to experience the qualities, like experiencing the feel of language production and reception, then one needs the embodied perceptions of the appropriate real objects.

Given the representational account of the inner-speech experience just sketched, BET also has an important explanatory benefit worth underscoring. Specifically, the fact that its key sensory representations are generated through perception rather than non-perceptual sources provides content that is better suited for the phenomenology of inner speech. To the point, recent research on perception versus imagination shows that confrontation with actual objects via perception enables the brain to collect enough data to create a stronger signal which passes a threshold from the merely imagined to a perceived reality (see Dijkstra and Fleming, 2023).

I submit that the perception of real incipient speech activities mixed with the perception of real sounds thus explains the *felt reality* of inner speech, that is, why the phenomenon is experienced as a real linguistic activity rather than experienced as a mere thought or reflection about that kind of activity—think of the difference between possessing a textbook description of how to produce the bilabial phoneme *P* in English (“put both lips together, voiceless exhale without vibration of the vocal cords, etc.”) versus having the experience of actually producing that phoneme. Inner speech is more like the latter, and BET’s representations through perception explain why.

Moreover, recent research on vision suggests that long-term memory stores a more fuzzy image with less fidelity when compared to the perception of objects from the current activation of the retina (Favila et al., 2022). So, normal perception is richer in detail than the normal memory of things that are no longer present, at least when they are directed at the same object, all things being equal. It is plausible that the same thing is true about the other sense modalities as well, including interoception. Thus, I submit that the role of genuine perceptual representations in the relevant sensory channels creates the *detailed phenomenology* associated with inner speech. For example, this is why one feels the directed movements of speaking, even fine-grained details regarding, for example, feeling the position and tensing at the lips for a “P” sound but not a “T” sound, if and when one is experiencing the pertinent phoneme during inner speech. One feels a real and detailed linguistic activity, not just a fuzzy thought about it.

### 6.2 Possible limits on the scope of BET

Given that BET aims to explain the central data for the experience of inner speech by grounding the phenomenal qualities of the language-like experiences in representations of actual objects and properties via the operations of the external perceptual systems, it follows that BET is restricted to those forms of inner speech with a language-like phenomenology. Are there kinds of inner speech that lack any language-like phenomenology? Some psychologists think so. The most well-known example is provided by Lev Vygotsky, who described an abstract non-phenomenal form of inner speech as a stage at the end of a developmental process wherein speaking a public language has not only become fully internalized but also transformed into pure thoughts about its meanings. As he put it metaphorically: “words die as they bring forth thought. Inner speech is to a large extent thinking in pure meanings” (Vygotsky, 1934/1986, p. 249).

Perhaps one can understand Vygotsky’s idea as follows: there is a transition from the experience of a full sentence to a condensed sentence with placeholders for words to the experience of a structure with only placeholders for words, a maximally condensed inner speech wherein the mind makes connections with meanings from a semantic network in a non-phenomenal way, with no pictures or images or felt impressions of words, just pure information.

As Alderson-Day and Fernyhough describe it: “The endpoint of the processes of transformation described by Vygotsky to accompany internalization is a stage of ‘thinking in pure meanings’ … in which all phenomenal properties of the language that transforms thinking are stripped away” (Alderson-Day and Fernyhough, 2014, p. 114).

The same is true for a similar idea described by Hurlburt, Heavey, and Kelsey as “unworded” inner speech. Hurlburt and colleagues say: “Sometimes (not frequently, we think) inner speaking is missing all of its words. That is, the person has the sense of innerly speaking (its production, its rhythm, etc.), and generally knows the sense of what is being said, but does not experience any words” (Hurlburt et al., 2013, p. 1482).^17^ The moral is that one should not expect BET to hold true for a supposed inner speech with no phenomenal linguistic properties since, it seeks to explain the phenomenal properties associated with the experience of inner speech. In fact, according to BET, the absence of represented sounds plus represented activities in the speech machinery is the reason *why* the phenomenal properties of a language have been stripped away, leaving only a form of pure thought about language.

There are other limits on the scope of what BET aims to explain. For example, I have not addressed the various triggers for inner speech and the differences they might make for the phenomena under consideration—different kinds of problem-solving tasks, silent reading tasks, even the open-ended conditions that exist when there is a random beep that occurs by the descriptive experience-sampling method (see text footnote 7). This much I leave for future research. Nevertheless, I think BET explains the central features of typical inner-speech experiences, as I discussed previously, including those discovered under the open-ended conditions for the descriptive experience-sampling method (see sec. 4.1).

### 6.3 Future directions

Let me close with some brief remarks about other possible variations on BET and extensions to topics other than inner speech. First, one might venture to explain the phenomenal aspects of inner speech by a more parsimonious BET-style hypothesis with only one kind of sensory input, say, utilizing just the interoception of the incipient speech activities. I call it BET-1. This hypothesis would still possess some of the advertised virtues of the full cross-modal BET: its key representations have actual and available objects as their referents, and they are perceptual representations that yield a felt reality and vividness of detail not afforded by representations from non-perceptual sources. Indeed, BET-1 might be confirmed over BET if, for example, neurophysical tests failed to detect the perception of sound during episodes of inner-speech experience. Unfortunately, I have little to say about the kind of illusion that would make BET-1 true, and BET’s cross-modal materials of incipient speech *plus* physical sounds come closer to the target illusory experience of speech sounds. So, I offer the cross-modal version as my best BET.

Second, BET might generalize in fruitful ways. For example, there is a similar phenomenon of “inner music” whereby people are able to hear and replay music, such as the finale of Stravinky’s *The Firebird*, “in one’s head” (for work on inner music, see Bailes, 2007; Cotter et al., 2019). I think it is worth exploring whether this kind of experience is likewise produced by things in and around the body, pace the generic BET. More specifically, I conjecture that such things as incipient movements for humming, rhythmic breaths, and biting one’s teeth could likewise feed into the mind’s constructive processes for perception, creating a similar cross-modal illusion of something that seems subjective and inside the mind. But, I leave further speculation for another occasion.

So, in summary, what is the experience of speaking and hearing an inner voice? By BET, this still small voice arises from perception in a mix of cross-modal sensory representations caused by actual physical objects in and around the body. For a few thousand years, maybe thousands of years, common folk and sages have thought that inner speech is entirely in the mind. But, if BET is correct, they were duped by one of nature’s greatest illusions.

## Data availability statement

The original contributions presented in the study are included in the article/supplementary material, further inquiries can be directed to the corresponding author.

## Author contributions

RE: Writing – review & editing, Writing – original draft.

## Footnotes

^1^Of course, there are qualifications. Normal individuals can experience their inner voice as an imitation of others, for example, when rehearsing a character for a play, or silently reading a character in a story (see Vilhauer, 2016). Obviously, when the system is damaged, as presumably in cases of schizophrenia, then the subjects may misidentify their inner voice as belonging to someone else (Hurlburt, 1990; Morin, 2009).

^2^Of course, inner hearing only applies to individuals with no hearing deficits. Hearing-impaired individuals experience visual imagery with their inner speech, not auditory imagery, as shown by effects on memory for the shape rather than the sound of letters (Wallace and Corballis, 1973) and effects for word length and hand movements in ASL (Wilson and Emmorey, 1998).

^3^A lesser number of studies have been concerned with how *frequently* subjects experience inner speech among those who do. A recent study by Christopher Heavey and colleagues determined the average over-estimates provided by their subjects to be roughly 68.3% of the time (Heavey et al., 2019). Thus, for many of those who experience it, inner speech is a regular phenomenon of waking life, even more than eating or thinking about sex.

^4^There are multiple channels for the external perception. As Andreas Lind and Robert Hartsuiker describe them: “auditory and bone conducted feedback reach the cochlea and allow us to hear our own voice, proprioceptive feedback gives us information about the position of the articulators, and tactile feedback gives us information about oral surfaces hitting each other” (Lind and Hartsuiker, 2020, p. 1).

^5^The current models just cited synthesize aspects of “comprehension” accounts whereby the system accesses information about the verbal output via general systems designed to comprehend language plus “production” models whereby the system accesses information about how the verbal output was produced. For reasons why Roelofs (2020) model is such a synthesis, see Nozari (2020).

^6^As is common in psycholinguistics, “lemma retrieval” maps a concept onto syntactic and semantic representations, and “word-form encoding” adds phonological information, both of which draw from a memory store not depicted but assumed.

^7^Briefly, the DES requires that subjects carry a beeper that, when it randomly sounds, is a prompt to write down notes regarding whatever inner experience they were having the moment before, followed by an interview with investigators who collaborate upon a “high-fidelity” apprehension of the experience (for a more complete description of the method and issues, see Hurlburt and Heavey, 2006; Hurlburt and Schwitzgebel, 2007; Hurlburt, 2011).

^8^This is the typical experience of inner speech. Yet psychologists have, for over a century, claimed to be able to sense their covert movements, even if guided by their own theories, for example, feeling the tension in the tongue and lips (see the citations in Sokolov, 1972; Loevensbruck et al., 2018).

^9^As the authors put it: “the duration of inspiration decreases and its velocity increases; conversely, the duration of expiration increases and the volume of air flow decreases dramatically” (Conrad and Schönle, 1979, p. 251). Moreover, although the authors of the study viewed the irregular pattern as an automatic process, breathing can be controlled, and the irregular pattern matches a controlled speech behavior—one normally gulps in air and then speaks with a longer and controlled exhale. Thus, one might conjecture that, with outer speech and inner speech too, an exhale sometimes marks the beginning of a sentence or separate clause, followed by a longer controlled phase for its completion. That is, the irregular breathing during inner speech is literally punctuated by concurrent speech processing commands that were copied and sent back into the inner loop. However, they were dampened for the external loop of covert speech activity.

^10^Consider that all kinds of “residual effects” are detected by the brain and even reach the level of conscious perception, for example, the residual effect of dizziness from medication for certain ailments, or the residual effect of fatigue from depression after therapy.

^11^In normal cases of inner speech, advocates of the deflationary interpretation would say that the feelings arise not from perception but from the predictions and expectations that arise from processing the efference copy of speech instructions. But in the experimental condition just described, there is no inner speech and hence no inner activities that correlate with it. Any feelings of activity in the speech musculature would then be due to its perception via interoceptive channels.

^12^As Ian Hacking once put it: “experimentation has a life of its own” (Hacking, 1983, p. 150), and hence, the results of older experiments that revealed incipient speech movements can be placed within quite different theoretical frameworks.

^13^The same kind of generic remarks are repeated in their chronological outline of what occurred during the experiment: “speech no longer possible. Can hear distinctly” (*ibid.* p. 4), “can feel pain pinprick” (*ibid.*, p. 5), and the “subject stated after recovery that he was ‘clear as a bell’ all this period” (*ibid.*, p. 6). Similarly, the authors go on to report in their summary that “the subject remained acutely conscious throughout the experiment and memory was not impaired” (*ibid.*, p. 7) and that “pain, touch, and other modalities of cutaneous sensation remained normal throughout” (*ibid.*, p. 8).

^14^Two points. First, I think BET is compatible with other causal theories, although I do not have the space to discuss them here. Second, I do not deny that the sensory representations have an internal component in addition to the external causal relation, for example, entering into inferential relations (see Block, 1998, for this kind of two-factor theory). But, BET is committed to the idea that the experience of inner speech is given by the referents via the causal component, with subsequent inferences having a more abstract, non-phenomenal character, as I go on to explain in the text.

^15^This is only an analogy. {gold mountain} is derived from {gold} and {mountain} by an operation in compositional semantics wherein the modifier {gold} indicates the kind of thing denoted by the noun {mountain}. But, {speech sounds} is built by a cross-modal perceptual mechanism wherein {incipient speech movements} is not a modifier that indicates the kind of sound designated by the noun {sound}—the incipient speech movements are silent, and the sounds are nonverbal.

^16^By saying that the components “ground” the experience, I mean something like the semantic notion implied by discussions of the “symbol grounding” problem in the computational literature (see Harnad, 1990). Thus, inner speech is not a phenomenon shared by disembodied computer systems with abstract processes over uninterpreted symbols. By BET, it requires a computational system imbued with experience and content, pacing the wide content of its sensory representations that denote actual objects and properties in and around the body that cause one’s perception of them. BET, thus, falls in line with other externalist approaches to mind and language as well as the general themes of embodied, embedded, enacted, and extended theories of cognition (Varela et al., 1991; Clark, 2008).

^17^Notice that the sense of “its production, its rhythm” implies a phenomenology, only one that does not arise from the experience of separate words. Nevertheless, I think this rhythm might be explained by a BET-like story about the corresponding sequence of incipient speech movements, but I do not have the space to pursue this here.
